# Alcohol consumption, genetic variants in the alcohol- and folate metabolic pathways and colorectal cancer risk: the JPHC Study

**DOI:** 10.1038/srep36607

**Published:** 2016-11-09

**Authors:** Thomas Svensson, Taiki Yamaji, Sanjeev Budhathoki, Akihisa Hidaka, Motoki Iwasaki, Norie Sawada, Manami Inoue, Shizuka Sasazuki, Taichi Shimazu, Shoichiro Tsugane

**Affiliations:** 1 Department of Global Health Policy, Graduate School of Medicine, The University of Tokyo, 7-3-1 Hongo, Bunkyo-ku, Tokyo 113-0033, Japan; 2 Epidemiology and Prevention Group, Center for Public Health Sciences, National Cancer Center, 5-1-1 Tsukiji, Chuo-ku, Tokyo 104-0045, Japan

## Abstract

The association between alcohol intake and colorectal cancer (CRC) may vary secondary to single nucleotide polymorphisms (SNPs) in two pathways related to alcohol intake. 375 cases of CRC were identified among 38 373 Japan Public Health Center-based prospective Study (JPHC Study) participants who had returned a baseline questionnaire, reported no diagnosis of any cancer and provided blood samples. For each case, two controls were selected on matching variables. Logistic regression models were used to determine matched Odds Ratios (OR) and 95% Confidence Intervals (CI) for the association between alcohol consumption, genetic polymorphisms of enzymes in the alcohol- and folate metabolic pathways (e.g. methylenetetrahydrofolate reductase (*MTHFR*) rs1801133) and CRC risk. Compared to never/occasional alcohol intake, moderate to heavy alcohol intake was associated with CRC (OR = 2.12, 95% CI, 1.34–3.36). When compared to the CC genotype, the *MTHFR* rs1801133 CT/TT genotype was inversely associated with CRC (OR = 0.72, 95% CI, 0.54–0.97). Never/occasional consumers of alcohol with the *MTHFR* rs1801133 CT/TT genotype were also at a reduced risk of CRC compared to never/occasional drinkers with the CC genotype (OR = 0.68, 95% CI, 0.47–0.98) (P for interaction = 0.27). The results indicate that the folate pathway is likely to be involved in alcohol-related CRC development.

Alcohol consumption is a well-known and established risk factor for colorectal cancer (CRC)^1^,^2^,^3^,^4^,^5^,^6^ with studies proposing a causal relation between alcohol consumption and CRC risk^4^,^7^. Alcohol differs from other dietary risk factors for CRC as it is suggested to increase CRC risk through two separate pathways: 1) the alcohol metabolic pathway which involves the oxidation of ethanol into acetaldehyde and subsequently acetate, resulting in cell exposure to the potentially carcinogenic metabolite acetaldehyde^8^, and 2) the folate metabolic pathway where alcohol acts as a folate antagonist resulting in folate deficiency^9^ which may lead to DNA hypo-methylation and aberrant DNA synthesis^10^. Conversely, high folate status such as high folate intake or low alcohol consumption are associated with a reduced risk of CRC^11^.

In addition to the above, single nucleotide polymorphisms (SNPs) may modify individual susceptibility to CRC by affecting important enzymes in either of the two metabolic pathways. E.g., in the alcohol metabolic pathway, aldehyde dehydrogenase-2 (*ALDH2*) rs671 polymorphisms result in enzymatic activity that is reduced (*ALDH2* rs671 GA genotype) or absent (*ALDH2* rs671 AA genotype) which in turn leads to the accumulation of, and increased exposure to acetaldehyde following alcohol intake^12^. In the folate metabolic pathway, 5,10-methylenetetrahydrofolate reductase (*MTHFR*) rs1801133 polymorphisms reduce MTHFR activity (*MTHFR* rs1801133 CT/TT genotype) and shift the available folate pool from DNA methylation to DNA synthesis, in particular in individuals with the *MTHFR* rs1801133 TT genotype and a concomitantly high folate status^13^.

Only limited information is available from prospective studies on the effect of genetic polymorphisms of enzymes in the alcohol metabolic pathway (alcohol dehydrogenase 1B (*ADH1B*) rs1229984, *ALDH2* rs671, cytochrome P450 2E1 (*CYP2E1*) rs3813867) and the folate metabolic pathway (*MTHFR* rs1801133, *MTHFR* rs1801131, methionine synthase (*MTR*) rs1805087, and methionine synthase reductase (*MTRR*) rs1801394) respectively, and their interaction with alcohol consumption. The aim of the current study was thus to investigate the effect of gene-alcohol interactions on CRC risk using a nested case-control study within the Japan Public Health Center-based prospective Study (JPHC Study). We hypothesize that genetic variants of alcohol- and folate metabolic enzymes will modify the association between alcohol intake and CRC risk as described in previous studies.

## Method

### Study population (including questionnaire survey and blood collection)

The JPHC Study^14^ is a large cohort with a baseline population of 140 420 individuals, and was conducted in two cohorts: Cohort I, aged 40–59, was initiated in 1990 and Cohort II, aged 40–69, was started in 1993. JPHC Study participants were identified by the population registries maintained by the local municipalities in 11 public health center (PHC) areas. The JPHC Study, including all methods described in the present study, has been approved by the Institutional Review Board of the National Cancer Center (approval number: 13-021) and the University of Tokyo (approval number: 10508), with reference to relevant ethical guidelines for medical research in Japan. Informed consent was obtained from each participant implicitly when they completed the baseline questionnaire, in which the purpose of the study and follow-up methods were described. Written information on the study was mailed to each participant and is published on the study web site ( http://epi.ncc.go.jp/jphc).

Study participants were asked to provide information through a self-administered questionnaire which included questions on personal and familial medical histories and lifestyle factors including smoking, alcohol consumption, physical exercise and dietary habits. Dietary habits were assessed through a food frequency questionnaire containing 44 items for Cohort I and 52 items for Cohort II. A total of 113 461 individuals returned the questionnaire. Additionally, collected at the time of health check-up (Cohort I 1990–1992; Cohort II: 1993–1995), 49 011 individuals donated 10-ml of venous blood drawn into vacutainer tubes containing heparin. Samples were divided into plasma and buffy layers, and preserved at −80 °C until analysis.

### Selection of cases and controls (including follow-up)

Cancer site and histology were coded using the International Classification of Diseases for Oncology, Third Edition (ICD-O-3)^15^. Details on the selection of cases and controls have been discussed elsewhere^16^. In brief, 375 cases of colorectal cancer (CRC) were identified up to 31 December 2003 through two sources: local major hospitals and population-based cancer registries. These cases occurred among a cohort of eligible subjects who had returned the baseline questionnaire, provided blood samples and reported no diagnosis of any cancer (n = 38 373 not including 13 ineligible subjects). All cases were pathologically confirmed as adenocarcinoma; 256 individuals were diagnosed with colon cancer (ICD-O-3: C180-C189) and 199 with rectal cancer (ICD-O-3: C199 and C209). For 370 of the 375 cases there was information about tumour depth; 120 tumours were of the intramucosal type and 250 of the invasive type.

Two controls were randomly selected from the cohort of 38 373 eligible subjects using incidence density sampling^17^. Controls, free of colorectal cancer history at the time when the case was diagnosed, were matched with each case on sex, age (within three years), date of blood drawn (within three months), time since last meal (within 4 hours) and study location (PHC area).

### Assessment of alcohol consumption

Alcohol consumption during the baseline survey was assessed using a validated self-administered food frequency questionnaire (FFQ). The method has been described in detail elsewhere^18^, and the validity of FFQ-based alcohol consumption estimations have been evaluated for both Cohort I and Cohort II in JPHC Study subsamples^19^,^20^. In brief, Cohort I and Cohort II differed in their assessment of alcohol consumption. For Cohort I, there were six categories of alcohol consumption (almost never, 1–3 days per month, 1–2 days per week, 3–4 days per week, 5–6 days per week, and every day). For those drinking at least once per week, additional information was requested regarding amount and type of alcoholic beverage consumed, in addition to providing a consumption frequency score: 1.5 (1–2 days per week), 3.5 (3–4 days per week), 5.5 (5–6 days per week), and 7 (daily).

For Cohort II, information on alcohol consumption status (never, former, or current) was obtained with details on alcohol consumption for former or current drinkers. Average consumption scores for Cohort II were: 1.5 (1–2 days per week), 3.5 (3–4 days per week), and 6 (almost daily).

Alcohol consumption was quantified in grams of ethanol by type of beverage consumed: 180 ml of shochu or awamori corresponded to 36 g of ethanol, 180 ml sake corresponded to 23 g, 633 ml of beer corresponded to 23 g, 30 ml of whiskey corresponded to 10 g, and 60 ml of wine corresponded to 6 g. Weekly ethanol intake was calculated by multiplying the frequency score with the quantity of alcohol consumed, and participants were categorised into one of three groups; never to occasional drinkers, <150, and ≥150 g of ethanol per week.

### Laboratory assay

To conduct genetic research within the framework of the JPHC Study, we obtained an additional approval from the institutional review board of the National Cancer Center (approval number: 2011-044), Tokyo, Japan, and provided all eligible subjects who donated a blood sample with the opportunity to refuse participation in the research. Genomic DNA was extracted from white blood cells in the buffy coat layer using a FlexiGene DNA kit (Qiagen, Hilden, Germany). Buffy coat samples were not available for all 16 pairs (i.e. 48 subjects) in one PHC area of Cohort II (Suita, Osaka). All but 12 buffy coat samples provided a sufficient amount of genomic DNA, and thus the following genotyping was performed among 356 cases and 709 controls. 46 SNPs including *ADH1B* rs1229984, *ALDH2* rs671, *CYP2E1* rs3813867, *MTHFR* rs1801133 (also known as *MTHFR* C677T), *MTHFR* rs1801131 (*MTHFR* A1298C), *MTRR* rs1801394 (*MTRR* A66G), and *MTR* rs1805087 (*MTR* A2756G ) were genotyped on the BioMark Dynamic Array platform (Fluidigm Corporation, South San Francisco, CA, USA) using the TaqMan SNP Genotyping Assays/Drug metabolism Genotyping Assays (Applied Biosystems, Foster City, CA) at GeneticLab, Hokkaido, Japan. Samples of cases and matched controls were genotyped in the same batch. All laboratory personnel were blinded with respect to case and control status.

### Statistical Analysis

Differences in baseline characteristics between cases and controls were determined using the Chi-square test for categorical variables and the Wilcoxon signed rank sum test for continuous variables. Conditional logistic regression models were used to determine matched Odds Ratios (OR) and 95% Confidence Intervals (CI) for the association between alcohol consumption, polymorphisms (*ADH1B* rs1229984, *ALDH2* rs671, *CYP2E1* rs3813867, *MTHFR* rs1801133, *MTHFR* rs1801131, *MTRR* rs1801394, and *MTR* rs1805087) and CRC risk. Model 1 (OR1) was matched for age (±3 years), sex, area, blood donation date (±2 months), and fasting time at blood donation (±5 hours). Model 2 (OR2) was further adjusted for smoking status, body mass index, family history of colorectal cancer, physical activity, and energy adjusted intake of red meat, processed meat, vegetables, fruits, fish, calcium, vitamin D, vitamin B2, vitamin B6, vitamin B12, and folate. Unconditional logistic regression models were used to determine the interaction between alcohol intake and polymorphisms of the alcohol- and folate- metabolic pathway in relation to the risk of CRC.

The Chi-square test was used to test for departures of the genotype distribution from the Hardy Weinberg equilibrium. Statistical analyses were performed using SAS (SAS software version 9.3; SAS Institute Inc., Cary, NC). The significance level was set as p < 0.05.

## Results

There were no statistically significant differences between cases and controls for any of the selected baseline characteristics (Table 1).

Alcohol intake was positively associated with colorectal cancer in both the minimally (OR = 1.66, 95% CI, 1.10–2.51) and fully adjusted conditional models (OR = 2.12, 95% CI, 1.34–3.36) (Table 2). In contrast, folate intake was not apparently associated with the risk of CRC.

All genotype frequencies (*ADH1B* rs1229984, *ALDH2* rs671, *CYP2E1* rs3813867, *MTHFR* rs1801133, *MTHFR* rs1801131, *MTRR* rs1801394, and *MTR* rs1805087) among controls were consistent with the Hardy Weinberg equilibrium (p > 0.05).

SNPs in the genes of alcohol-metabolic enzymes (*ADH1B* rs1229984, *ALDH2* rs671, and *CYP2E1* rs3813867) were not significantly associated with CRC risk (Table 2). Among SNPs in the genes of folate-metabolic enzymes, the *MTHFR* rs1801133 CT/TT genotype showed a reduced risk of CRC (OR = 0.72, 95% CI, 0.54–0.97) compared to the *MTHFR* rs1801133 CC genotype. The inverse association was significant in individuals with the *MTHFR* rs1801133 CT genotype (OR = 0.66, 95% CI, 0.48–0.91), while non-significant in those with the *MTHFR* rs1801133 TT genotype (OR = 0.87, 95% CI, 0.58–1.30).

We did not observe any interaction between alcohol intake and SNPs in the genes of alcohol- and folate metabolic enzymes in relation to the risk of CRC (p > 0.05, Table 3). In general, increased alcohol intake was positively associated with the risk of CRC irrespective of genotype. Of interest, among never/occasional drinkers, the *MTHFR* rs1801133 CT/TT genotype was related to a decreased risk of CRC (OR = 0.68, 95% CI, 0.47–0.98) compared to the *MTHFR* rs1801133 CC genotype. More precisely, the corresponding ORs were 0.71 (95% CI, 0.48–1.05) and 0.60 (95% CI, 0.36–1.01) for the *MTHFR* rs1801133 CT and TT genotype respectively. When further stratified by folate intake, we observed a similarly but non-significantly decreased risk among never/occasional drinkers with the *MTHFR* rs1801133 CT/TT genotype irrespective of their folate intake levels (Supplementary Table S1).

Sensitivity analyses excluding cases in the first two years of follow-up yielded similar results for Tables 2 and 3 (data not shown).

## Discussion

Our findings show that moderate to heavy alcohol consumption increases the risk of CRC, whereas the *MTHFR* rs1801133 CT/TT genotype is inversely associated with CRC risk. Moreover, individuals with low alcohol consumption and the *MTHFR* rs1801133 CT/TT genotype were at a significantly reduced risk of CRC, while those with high alcohol consumption and the *MTHFR* rs1801133 CC genotype were at a significantly increased risk.

Our result of a potentially protective effect of the *MTHFR* rs1801133 CT/TT genotype on CRC is in accordance with previous findings^21^,^22^,^23^,^24^. The primary function of MTHFR is to catalyse the conversion of 5,10-methylenetetrahydrofolate to 5-methyltetrahydrofolate^25^. Individuals with the *MTHFR* rs1801133 CT/TT genotype are known to have the enzyme with a decreased activity, and likely have accumulated levels of 5,10-methylenetetrahydrofolate. Increased levels of 5,10-methylenetetrahydrofolate would allow for increased DNA synthesis^1^, and may thereby reduce the risk of CRC. Although it has been suggested that the *MTHFR* rs1801133 CT and TT genotype are related to a 35% and 70% reduction in the enzyme activity respectively^26^, we did not observe any apparent difference in a reduced risk of CRC between individuals with the CT and TT genotype.

We failed to find any associations between the *MTHFR* rs1801131 polymorphism and CRC. Previous studies have reported both an inverse and positive association between the *MTHFR* rs1801131 CC genotype and colon cancer^27^ and CRC^28^ respectively when compared to the *MTHFR* rs1801131 AA/AC genotype at low folate or high alcohol intake levels. Possible reasons for the discrepant results could be differences in end points between studies (i.e. colon cancer vs CRC) or the small sample sizes used^28^. In addition, our study could not find any association between *MTR* rs1805087 or *MTRR* rs1801394 polymorphisms and CRC risk.

Although some findings suggest that variant alleles in the genes of alcohol-metabolic enzymes are the main culprits of alcohol-related CRC^29^,^30^,^31^,^32^,^33^, other findings have instead shown that genetic variants of alcohol metabolic enzymes do not increase CRC risk^34^,^35^ and do not modify the effect of alcohol on CRC^5^,^36^. Our own findings show that the associations of *ADH1B* rs1229984, and *CYP2E1* rs3813867 polymorphisms with CRC remain close to one whereas *ALDH2* rs671 polymorphisms may even be inversely associated with CRC. A possibly protective effect of *ALDH2* rs671 polymorphism is counter-intuitive due to the potentially carcinogenic effects of acetaldehyde^8^, but the frequency of daily drinkers among those with the *ALDH2* rs671 AA genotype in the current study was very low (n = 2) which prevents us from drawing any conclusion from these results.

There are several limitations that need to be mentioned: Health check-up participants have healthier lifestyle habits compared to their counterparts^37^ which could lead to an underestimation of our results. We grouped minor allele homozygotes with minor allele heterozygotes which could lead to further underestimation of results. Finally, results may not be generalizable to other populations.

Despite such limitations, this study has a major strength: due to the prospective nature of the study, all data was collected before development of disease thereby minimizing recall bias and strengthening any associations found between predictors and outcome.

In summary, alcohol consumption is positively associated with CRC, whereas the *MTHFR* rs1801133 CT/TT genotype is inversely associated with CRC risk. Furthermore, low grade alcohol consumption together with the *MTHFR* rs1801133 CT/TT genotype reduces CRC risk whereas high grade alcohol consumption together with the *MTHFR* rs1801133 CC genotype increases CRC risk. These findings indicate that the folate metabolic pathway is likely to be involved in alcohol-related CRC development.

## Additional Information

**How to cite this article**: Svensson, T. *et al.* Alcohol consumption, genetic variants in the alcohol- and folate metabolic pathways and colorectal cancer risk: the JPHC Study. *Sci. Rep.* **6**, 36607; doi: 10.1038/srep36607 (2016).

**Publisher’s note**: Springer Nature remains neutral with regard to jurisdictional claims in published maps and institutional affiliations.

## Supplementary Material

Supplementary Information

## Figures and Tables

**Table 1:** Baseline characteristics of cases and controls.

Characteristics	Cases	Controls	*P* value^a^
*n*	356	709	
Age, mean (SD)	56.7 (7.32)	56.6 (7.18)	Matching value
Men (%)	183 (51.4)	366 (51.6)	Matching value
Smoking status	200 (56.5)	434 (61.4)	0.45
Never (%)	59 (16.7)	106 (15.0)	
Former (%)	44 (12.4)	72 (10.2)	
Current: <30 pack years (%)	51 (14.4)	95 (13.4)	
Current: ≥30 pack years (%)	200 (56.5)	434 (61.4)	
Alcohol consumption			0.18
Never to occasional (%)	200 (57.8)	423 (62.1)	
Daily and <150 g/week (%)	41 (11.9)	88 (12.9)	
Daily and ≥150 g/week (%)	105 (30.4)	170 (25.0)	
BMI (kg/m^2^), mean (SD)	23.7 (3.0)	23.5 (2.9)	0.32
Physical activity (%)	74 (20.9)	117 (16.8)	0.11
Total energy intake (kcal/day), mean (SD)	1683 (582)	1690 (642)	0.77
Red meat intake (g/day), mean (SD)	14.8 (9.6)	14.3 (10.8)	0.13
Processed meat intake (g/day), mean (SD)	3.3 (3.7)	3.3 (3.6)	0.84
Fish intake (g/day), mean (SD)	53.5 (31.3)	52.2 (33.1)	0.34
Vegetable intake (g/day), mean (SD)	102.9 (90.9)	95.5 (76.3)	0.41
Fruit intake (g/day), mean (SD)	95.8 (69.0)	96.3 (80.3)	0.54
Calcium (mg/day), mean (SD)	437.4 (201.9)	425.2 (188.8)	0.54
Vitamin D (μg/day), mean (SD)	5.7 (2.7)	5.5 (2.8)	0.36
Vitamin B2 (mg/day), mean (SD)	1.2 (0.4)	1.2 (0.4)	0.25
Vitamin B6 (mg/day), mean (SD)	1.0 (0.2)	1.0 (0.2)	0.78
Vitamin B12 (μg/day), mean (SD)	6.4 (3.4)	6.3 (3.2)	0.95
Folate (μg/day), mean (SD)	305.2 (100.9)	302.3 (99.9)	0.66
Family history of colorectal cancer (%)	7 (1.1)	8 (2.0)	0.27

^a^Based on chi-square test or Wilcoxon signed rank sum test.

**Table 2:** Association between alcohol consumption, *ADH1B*, *ALDH2*, *CYP2E1*, *MTHFR*, *MTRR*, *and MTR* polymorphisms, and colorectal cancer.

	Genotype Frequency (%)	Cases (*n*)/Controls (*n*)	OR1 (95% CI)^a^	OR2 (95% CI)^b^
Alcohol consumption				
Never to occasional		200/423	1.00 (Reference)	1.00 (Reference)
Daily and <150 g/week		41/88	1.13 (0.71–1.81)	1.25 (0.76–2.04)
Daily and ≥150 g/week		105/170	**1**.66* (**1**.**10**–**2**.**51**)	**2**.12** (**1**.**34**–**3**.**36**)
P for trend				0.002
Energy adjusted folate intake^c^				
Quartile 1		99/187	1.00 (Reference)	1.00 (Reference)
Quartile 2		104/179	0.88 (0.61–1.28)	0.89 (0.57–1.40)
Quartile 3		84/173	0.91 (0.63–1.31)	0.91 (0.55–1.49)
Quartile 4		90/174	0.96 (0.67–1.38)	0.88 (0.46–1.67)
P for trend				0.74
**Alcohol Metabolic Pathway**				
*ADH1B* rs1229984				
AA	56.5	194/400	1.00 (Reference)	1.00 (Reference)
AG	36.3	139/257	1.10 (0.85–1.43)	1.16 (0.87–1.55)
GG	7.2	23/51	0.93 (0.55–1.58)	1.05 (0.59–1.87)
AG + GG	43.5	162/308	1.07 (0.84–1.38)	1.15 (0.87–1.51)
*ALDH2* rs671				
GG	65.5	248/464	1.00 (Reference)	1.00 (Reference)
GA	31.4	96/222	0.82 (0.62–1.08)	0.88 (0.63–1.21)
AA	3.1	12/22	1.02 (0.50–2.08)	0.90 (0.42–1.95)
GA + AA	34.5	108/244	0.84 (0.64–1.10)	0.88 (0.64–1.20)
*CYP2E1* rs3813867				
GG	58.1	214/411	1.00 (Reference)	1.00 (Reference)
CG	36.0	113/255	0.84 (0.63–1.12)	0.88 (0.64–1.21)
CC	5.9	29/42	1.34 (0.81–2.21)	1.43 (0.82–2.50)
CG + CC	42.0	142/297	0.92 (0.70–1.19)	0.96 (0.72–1.29)
**Folate Metabolic Pathway**				
*MTHFR* rs1801133				
CC	36.2	148/256	1.00 (Reference)	1.00 (Reference)
CT	47.3	148/335	0.75 (0.57–1.002)	**0**.66* (**0**.**48**–**0**.**91**)
TT	16.5	60/117	0.89 (0.61–1.28)	0.87 (0.58–1.30)
CT + TT	63.8	208/452	0.79 (0.61–1.03)	**0**.72* (**0**.**54**–**0**.**97**)
*MTHFR* rs1801131				
AA	63.8	238/452	1.00 (Reference)	1.00 (Reference)
AC	32.3	100/229	0.82 (0.61–1.10)	0.80 (0.58–1.11)
CC	3.8	18/27	1.27 (0.68–2.36)	1.63 (0.82–3.22)
AC + CC	36.2	118/256	0.87 (0.66–1.15)	0.88 (0.65–1.20)
*MTRR* rs1801394				
AA	50.9	186/360	1.00 (Reference)	1.00 (Reference)
GA	41.2	133/292	0.88 (0.67–1.16)	0.88 (0.66–1.19)
GG	7.9	37/56	1.29 (0.82–2.04)	1.45 (0.88–2.38)
GA + GG	49.2	170/348	0.95 (0.73–1.22)	0.97 (0.73–1.28)
*MTR* rs1805087				
AA	63.6	217/450	1.00 (Reference)	1.00 (Reference)
GA	32.5	127/230	1.16 (0.88–1.52)	1.10 (0.82–1.49)
GG	4.0	12/28	0.90 (0.45–1.82)	0.87 (0.39–1.95)
GA + GG	36.4	139/258	1.13 (0.86–1.48)	1.08 (0.81–1.45)

Based on conditional logistic regression models.

*p ≤ 0.05, **p ≤ 0.01.

^a^Matched for age (±3 years), sex, area, blood donation date (±2 months), and fasting time at blood donation (±5 hours).

^b^Further adjusted for body mass index, family history of colorectal cancer, physical activity, energy adjusted intake of red meat, processed meat, vegetables, fruits, fish, energy adjusted intake of calcium, vitamin D, vitamin B2, vitamin B6, vitamin B12, folate, and smoking status.

^c^Further adjusted for body mass index, family history of colorectal cancer, physical activity, energy adjusted intake of red meat, processed meat, vegetables, fruits, fish, energy adjusted intake of calcium, vitamin D, vitamin B2, vitamin B6, vitamin B12, and smoking status.

**Table 3:** Colorectal cancer risk associated with alcohol consumption according to polymorphisms.

	AA			AG + GG			*P* for Interaction^b^
	Cases (*n*)/Controls (*n*)	OR1 (95% CI)^a^	OR2 (95% CI)^b^	Cases (*n*)/Controls (*n*)	OR1 (95% CI)^a^	OR2 (95% CI)^b^	
***ADH1B* rs1229984**							
Alcohol consumption							
Never to occasional	104/237	1.00 (Reference)	1.00 (Reference)	96/185 (84 + 12/148 + 37)	1.20 (0.85–1.68)	1.24 (0.87–1.76)	
Daily and <150 g/week	24/57	1.12 (0.63–1.99)	1.15 (0.64–2.09)	17/31 (15 + 2/28 + 3)	1.52 (0.76–3.00)	1.53 (0.75–3.10)	
Daily and ≥150 g/week	60/86	**2**.03** (**1**.**24**–**3**.**35**)	**2**.34** (**1**.**37**–**3**.**99**)	45/84 (36 + 9/73 + 11)	1.54 (0.92–2.59)	**1**.76* (**1**.**01**–**3**.**06**)	0.25
***ALDH2*** **rs671**							
	**GG**			**GA + AA**			
Alcohol consumption							
Never to occasional	116/242	1.00 (Reference)	1.00 (Reference)	84/180 (73 + 11/159 + 21)	1.04 (0.73–1.49)	1.01 (0.70–1.46)	
Daily and <150 g/week	30/54	1.37 (0.78–2.41)	1.41 (0.79–2.52)	11/34 (10 + 1/33 + 1)	0.85 (0.39–1.86)	0.77 (0.34–1.77)	
Daily and ≥150 g/week	94/147	**1**.72* (**1**.**07**–**2**.**78**)	**1**.94* (**1**.**17**–**3**.**22**)	11/23 (11 + 0/23 + 0)	1.31 (0.57–3.01)	1.32 (0.55–3.20)	0.34
***CYP2E1*** **rs3813867**							
	**GG**			**CG + CC**			
Alcohol consumption							
Never to occasional	120/245	1.00 (Reference)	1.00 (Reference)	80/177 (66 + 14/149 + 28)	0.92 (0.65–1.30)	1.00 (0.70–1.43)	
Daily and <150 g/week	27/52	1.27 (0.72–2.25)	1.29 (0.72–2.32)	14/36 (12 + 2/29 + 7)	0.92 (0.46–1.84)	0.99 (0.48–2.04)	
Daily and ≥150 g/week	61/96	**1**.65* (**1**.**01**–**2**.**68**)	**1**.86* (**1**.**11**–**3**.**14**)	44/74 (32 + 12/70 + 4)	1.55 (0.92–2.61)	**1**.87* (**1**.**07**–**3**.**27**)	0.83
***MTHFR*** **rs1801133**							
	**CC**			**CT + TT**			
Alcohol consumption							
Never to occasional	86/151	1.00 (Reference)	1.00 (Reference)	114/271 (84 + 30/190 + 81)	0.73 (0.52–1.03)	**0**.68* (**0**.**47**–**0**.**98**)	
Daily and <150 g/week	12/30	0.82 (0.38–1.77)	0.72 (0.32–1.64)	29/58 (21 + 8/45 + 13)	1.02 (0.57–1.81)	1.02 (0.57–1.83)	
Daily and ≥150 g/week	45/60	1.68 (0.96–2.94)	**1**.91* (**1**.**05**–**3**.**45**)	60/110 (40 + 20/90 + 20)	1.18 (0.71–1.97)	1.27 (0.74–2.17)	0.27
***MTHFR*** **rs1801131**							
	**AA**			**AC + CC**			
Alcohol consumption							
Never to occasional	127/266	1.00 (Reference)	1.00 (Reference)	73/156 (61 + 12/137 + 19)	0.98 (0.69–1.40)	0.97 (0.67–1.39)	
Daily and <150 g/week	28/53	1.32 (0.75–2.31)	1.36 (0.76–2.42)	13/35 (11 + 2/35 + 3)	0.90 (0.44–1.84)	0.82 (0.38–1.77)	
Daily and ≥150 g/week	75/114	**1**.74* (**1**.**10**–**2**.**75**)	**1**.96** (**1**.**20**–**3**.**20**)	30/56 (26 + 4/53 + 3)	1.42 (0.80–2.55)	1.55 (0.83–2.90)	0.56
***MTRR*** **rs1801394**							
	**AA**			**GA + GG**			
Alcohol consumption							
Never to occasional	96/224	1.00 (Reference)	1.00 (Reference)	104/198 (84 + 20/169 + 20)	1.23 (0.88–1.73)	1.13 (0.79–1.60)	
Daily and <150 g/week	22/34	1.77 (0.94–3.33)	1.77 (0.93–3.36)	19/54 (13+6/44 + 10)	0.96 (0.52–1.80)	0.88 (0.46–1.70)	
Daily and ≥150 g/week	59/90	**1**.93* (**1**.**17**–**3**.**17**)	**2**.00* (**1**.**17**–**3**.**41**)	46/80 (35 + 11/65 + 15)	**1**.70* (**1**.**00**–**2**.**88**)	**1**.90* (**1**.**09**–**3**.**33**)	0.17
***MTR*** **rs1805087**							
	**AA**			**GA + GG**			
Alcohol consumption							
Never to occasional	124/274	1.00 (Reference)	1.00 (Reference)	76/148 (66 + 10/132 + 16)	1.12 (0.79–1.60)	1.12 (0.78–1.61)	
Daily and <150 g/week	27/48	1.45 (0.82–2.56)	1.43 (0.79–2.58)	14/40 (14 + 0/34 + 6)	0.91 (0.46–1.81)	0.95 (0.47–1.92)	
Daily and ≥150 g/week	62/111	1.56 (0.97–2.50)	**1**.79* (**1**.**07**–**3**.**00**)	43/59 (42 + 1/54 + 5)	**2**.00* (**1**.**18**–**3**.**40**)	**2**.15** (**1**.**22**–**3**.**78**)	0.46

Based on unconditional logistic regression models.

*p ≤ 0.05, **p ≤ 0.01.

^a^Adjusted for age (continuous), sex, area, blood donation date (season), and fasting time at blood donation (±5 hours).

^b^Further adjusted for body mass index, family history of colorectal cancer, physical activity, energy adjusted intake of red meat, processed meat, vegetables, fruits, fish, energy adjusted intake of calcium, vitamin D, vitamin B2, vitamin B6, vitamin B12, folate, and smoking status.
