# Supplementary material for: Alcohol consumption, genetic variants in the alcohol- and folate metabolic pathways and colorectal cancer risk: the JPHC Study
Source: Sci Rep. 2016 Nov 9;6:36607. doi: 10.1038/srep36607 (PMC5101526; doi:10.1038/srep36607)
Supplement: Supplementary Information [file srep36607-s1.pdf]

Alcohol consumption, genetic variants in the alcohol- and folate metabolic pathways and colorectal cancer risk: the JPHC Study

Thomas Svensson, MD[1,2], Taiki Yamaji, MD, PhD[2], Sanjeev Budhathoki, PhD[2], Akihisa Hidaka, MD[2], Motoki Iwasaki, MD, PhD[2], Norie Sawada, MD, PhD[2], Manami Inoue, MD, PhD[1,2], Shizuka Sasazuki, MD, PhD[2], Taichi Shimazu, MD, PhD[2], and Shoichiro Tsugane, MD, DMSc[2]

1. Department of Global Health Policy, Graduate School of Medicine, The University of Tokyo, 7-3-1 Hongo, Bunkyo-ku, Tokyo 113-0033 Japan, Thomas Svensson PhD Candidate.
2. Epidemiology and Prevention Group, Center for Public Health Sciences, National Cancer Center, 5-1-1 Tsukiji, Chuo-ku, Tokyo 104-0045, Japan, Thomas Svensson visiting researcher, Taiki Yamaji section head, Sanjeev Budhathoki research resident, Akihisa Hidaka research resident, Motoki Iwasaki chief, Norie Sawada section head, Manami Inoue project scientist, Shizuka Sasazuki chief, Taichi Shimazu section head, Shoichiro Tsugane director.

Appendix

Members of the Japan Public Health Center-based Prospective Study (JPHC Study, principal investigator: S. Tsugane) Group are: S. Tsugane, N. Sawada, M. Iwasaki, S. Sasazuki, T. Yamaji, T. Shimazu and T. Hanaoka, National Cancer Center, Tokyo; J. Ogata, S. Baba, T. Mannami, A. Okayama, and Y. Kokubo, National Cerebral and

Cardiovascular Center, Osaka; K. Miyakawa, F. Saito, A. Koizumi, Y. Sano, I. Hashimoto, T. Ikuta, Y. Tanaba, H. Sato, Y. Roppongi, T. Takashima and H. Suzuki, Iwate Prefectural Ninohe Public Health Center, Iwate; Y. Miyajima, N. Suzuki, S. Nagasawa, Y. Furusugi, N. Nagai, Y. Ito, S. Komatsu and T. Minamizono, Akita Prefectural Yokote Public Health Center, Akita; H. Sanada, Y. Hatayama, F. Kobayashi, H. Uchino, Y. Shirai, T. Kondo, R. Sasaki, Y. Watanabe, Y. Miyagawa, Y. Kobayashi, M. Machida, K. Kobayashi and M. Tsukada, Nagano Prefectural Saku Public Health Center, Nagano; Y. Kishimoto, E. Takara, T. Fukuyama, M. Kinjo, M. Irei, and H. Sakiyama, Okinawa Prefectural Chubu Public Health Center, Okinawa; K. Imoto, H. Yazawa, T. Seo, A. Seiko, F. Ito, F. Shoji and R. Saito, Katsushika Public Health Center, Tokyo; A. Murata, K. Minato, K. Motegi, T. Fujieda and S. Yamato, Ibaraki Prefectural Mito Public Health Center, Ibaraki; K. Matsui, T. Abe, M. Katagiri, M. Suzuki, K. and Matsui, Niigata Prefectural Kashiwazaki and Nagaoka Public Health Center, Niigata; M. Doi, A. Terao, Y. Ishikawa, and T. Tagami, Kochi Prefectural Chuo-higashi Public Health Center, Kochi; H. Sueta, H. Doi, M. Urata, N. Okamoto, F. Ide, H. Goto and R Fujita, Nagasaki Prefectural Kamigoto Public Health Center, Nagasaki; H. Sakiyama, N. Onga, H. Takaesu, M. Uehara, T. Nakasone and M. Yamakawa, Okinawa Prefectural Miyako Public Health Center, Okinawa; F. Horii, I. Asano, H. Yamaguchi, K. Aoki, S. Maruyama, M. Ichii, and M. Takano, Osaka Prefectural Suita Public Health Center, Osaka; Y. Tsubono, Tohoku University, Miyagi; K. Suzuki, Research Institute for Brain and Blood Vessels Akita, Akita; Y. Honda, K. Yamagishi, S. Sakurai and N. Tsuchiya, University of Tsukuba, Ibaraki; M. Kabuto, National Institute for Environmental Studies, Ibaraki; M. Yamaguchi, Y. Matsumura, S. Sasaki, and S. Watanabe, National Institute of Health and Nutrition, Tokyo; M. Akabane, Tokyo University of Agriculture, Tokyo; T. Kadowaki and M. Inoue, The University of Tokyo, Tokyo; M. Noda and T. Mizoue, National Center for Global Health and Medicine, Tokyo; Y. Kawaguchi, Tokyo Medical and Dental University,

Tokyo; Y. Takashima and Y. Yoshida, Kyorin University, Tokyo; K. Nakamura and R. Takachi, Niigata University, Niigata; J. Ishihara, Sagami Women's University, Kanagawa; S. Matsushima and S. Natsukawa, Saku General Hospital, Nagano; H. Shimizu, Sakihae Institute, Gifu; H. Sugimura, Hamamatsu University School of Medicine, Shizuoka; S. Tominaga, Aichi Cancer Center, Aichi; N. Hamajima, Nagoya University, Aichi; H. Iso and T. Sobue, Osaka University, Osaka; M. Iida, W. Ajiki, and A. Ioka, Osaka Medical Center for Cancer and Cardiovascular Disease, Osaka; S. Sato, Chiba Prefectural Institute of Public Health, Chiba; E. Maruyama, Kobe University, Hyogo; M. Konishi, K. Okada, and I. Saito, Ehime University, Ehime; N. Yasuda, Kochi University, Kochi; S. Kono, Kyushu University, Fukuoka; S. Akiba, Kagoshima University, Kagoshima; T. Isobe, Keio University, Tokyo; Y. Sato, Tokyo Gakugei University, Tokyo.
